# Prediction of Hospital Mortality in Patients with ST Segment Elevation Myocardial Infarction: Evolution of Risk Measurement Techniques and Assessment of Their Effectiveness (Review)

**DOI:** 10.17691/stm2024.16.4.07

**Published:** 2024-08-30

**Authors:** B.I. Geltser, I.G. Domzhalov, K.I. Shakhgeldyan, N.S. Kuksin, E.A. Kokarev, R.L. Pak, V.N. Kotelnikov

**Affiliations:** MD, DSc, Professor, Corresponding Member of the Russian Academy of Science, Deputy Director for Science of the School of Medicine and Life Sciences; Far Eastern Federal University, 10 Village Ayaks, Island Russkiy, Vladivostok, 690922, Russia; PhD Student, School of Medicine and Life Sciences; Far Eastern Federal University, 10 Village Ayaks, Island Russkiy, Vladivostok, 690922, Russia; Physician, Intensive Care Department, Regional Vascular Surgery Center; Primorsky Regional Clinical Hospital No.1, 57 Aleutskaya St., Vladivostok, 690091, Russia; DSc, Associate Professor, Director of the Institute of Information Technologies; Vladivostok State University, 41 Gogolya St., Vladivostok, 690014, Russia; Head of Laboratory for Big Data Analysis in Medicine and Healthcare, School of Medicine and Life Sciences; Far Eastern Federal University, 10 Village Ayaks, Island Russkiy, Vladivostok, 690922, Russia; PhD Student, Institute of Mathematics and Computer Technology; Far Eastern Federal University, 10 Village Ayaks, Island Russkiy, Vladivostok, 690922, Russia; Research Assistant, Laboratory for Big Data Analytics in Medicine and Healthcare, School of Medicine and Life Sciences; Far Eastern Federal University, 10 Village Ayaks, Island Russkiy, Vladivostok, 690922, Russia; MD, PhD, Head of the Intensive Care Department, Regional Vascular Surgery Center; Far Eastern Federal University, 10 Village Ayaks, Island Russkiy, Vladivostok, 690922, Russia; Physician, Intensive Care Department, Regional Vascular Surgery Center; Far Eastern Federal University, 10 Village Ayaks, Island Russkiy, Vladivostok, 690922, Russia; MD, DSc, Professor, Department of Clinical Medicine, School of Medicine and Life Sciences; Far Eastern Federal University, 10 Village Ayaks, Island Russkiy, Vladivostok, 690922, Russia

**Keywords:** ST segment elevation myocardial infarction, hospital mortality, prognostic models, machine learning

## Abstract

Risk stratification of hospital mortality in patients with ST segment elevation myocardial infarction on the electrocardiogram is an important part of the specialized medical care provision. The systematic review presents scientific literature data characterizing the predictive value of both classical prognostic scales (GRACE, CADDILLAC, TIMI risk score for STEMI, RECORD, etc.) and new risk measurement tools developed on the basis of modern machine learning techniques. Most studies on this issue are often focused on the search for new predictors of adverse events, which allow to detail the relations between indicators of the clinical and functional status of patients and the end point of the study. Here, an important task is to develop hospital mortality prognostic algorithms characterized by explainable artificial intelligence and trusted by doctors.

## Introduction

Coronary heart disease (CHD) is the most spread cause of disability and mortality in most countries worldwide. ST segment elevation myocardial infarction on the electrocardiogram (STEMI) is the most severe clinical form of CHD and is associated with a high risk of adverse outcomes, including hospital mortality (HM). In the Russian Federation, the HM rate for STEMI varies within 13–14%, which is comparable to European countries and indicates the need to improve risk measurement tools that allow timely assess the likelihood of adverse events [1]. To assess the risk of HM in patients with STEMI, there were over 50 scales and prognostic algorithms developed in different countries, some of which are recommended by professional communities for common use and proved their effectiveness in real clinical practice [2–61]. Such scales include GRACE (Global Registry of Acute Coronary Events), CADILLAC (Controlled Abciximab and Device Investigation to Lower Late Angioplasty Complications), TIMI risk score for STEMI (Thrombolysis in Myocardial Infarction risk score for ST segment elevation myocardial infarction), RECORD, and etc. [3–6]. Currently, the research is ongoing with the aim to find new HM predictors, which add-up to the structure of earlier developed “classical” scales to increase the prediction accuracy. Also, constant expansion of STEMI patient registries requires modern machine learning (ML) techniques to process and analyze big data. This brings up new knowledge that provides details on the relations of potential predictors to the study end point. Predictive algorithms based on ML techniques are increasingly used to assess the risk of adverse events in various areas of clinical medicine, thus their quality improvement is the subject of multiple studies.

**The aim of this review** is to analyze scientific publications on prediction of hospital mortality in patients with STEMI and to assess the possibility to improve risk measurement tools based on modern machine learning techniques.

## Literature sources

Literature sources were searched in the PubMed (MEDLINE), Web of Science, Scopus, eLIBRARY.RU and Cochrane Database of Systematic Reviews databases using the following key words: “ST segment elevation myocardial infarction and mortality”, “ST segment elevation myocardial infarction and prognostic scales”, “ST segment elevation myocardial infarction and prognosis”, “scale prognosis of in-hospital mortality and TEMI”, “ST segment elevation myocardial infarction and prognosis scale in hospital mortality”. Based on the query results, 46,134 documents were selected. Duplicates were removed and the following documents were excluded: the documents that did not contain the data required for analysis (techniques to develop predictive models, indicators of their accuracy, information about predictors); the freely available documents with full-text materials; and studies with insufficient sample size (<200). The final version of the systematic review included 102 documents published during the period from 1999 to 2024. The most cited documents are provided in Tables 1 and 2.

**T a b l e 1:** Analysis of the predictive accuracy of classical models of hospital mortality in patients with STEMI

Model	Sample size	ML technique	Predictors	Quality metrics		
				AUC	Sen	Sp
PREDICT, 1999 [7]	6134	MLR	Age, BUN, Charlson comorbidity index, CS, congestive cardiac failure, history of cardiovascular disease, ECG data	0.79	—	—
TIMI risk score for STEMI, 2000 [5]	15,078	MLR	Age, AHF class (T. Killip class), HR, SBP, body weight, DM 2, AH, history of angina pectoris, time to revascularization >4 h, anterior MI	0.784	—	—
GUSTO, 2000 [8]	41,021	CR	Age, HR, LVEF, history of MI, CHF events, or pulmonary edema during hospitalization	0.8	—	—
PAMI, 2004 [9]	3252	MLR	Age, AHF class (T. Killip class), HR, DM 2, anterior MI	0.784	—	—
Zwolle, 2004 [10]	1791	MLR	Age, AHF class (T. Killip class), blood flow according to TIMI, TVCAD, time to revascularization >4 h, anterior MI	0.902	—	—
CADILLAC, 2005 [4]	2982	MLR	Age, AHF class (T. Killip class), GFR, LVEF, blood flow according to TIMI (0–2), Ht, TVCAD	0.83	—	—
GRACE, 2006 [3]	43,810	CR	Age, HR, SBP, AHF class (T. Killip class), Cr, cardiac arrest at the time of admission, ST segment elevation, diagnostically significant increase in the level of heart-specific enzymes	0.83	—	—
RECORD, 2010 [6]	796	MLR	Age, AHF class (T. Killip class), SBP, DM 2, ST segment elevation, Hb	0.856	0.785	0.785

N o t e: AH — arterial hypertension, AHF — acute heart failure, AUC — area under the ROC curve, BUN — blood urea level, CHF — chronic heart failure, CR — Cox regression, Cr — creatinine, CS — cardiogenic shock, DM 2 — diabetes mellitus type 2, ECG — electrocardiogram, GFR — glomerular filtration rate, Hb — hemoglobin, HR — heart rate, Ht — hematocrit, LVEF — left ventricular ejection fraction, MI — myocardial infarction, ML — machine learning, MLR — multivariate logistic regression, SBP — systolic blood pressure, Sen — sensitivity, Sp — specificity, STEMI — ST segment elevation myocardial infarction, TIMI — coronary blood flow assessment scale, TVCAD — three-vessel coronary artery disease.

**T a b l e 2:** Analysis of prognostic accuracy of new hospital mortality risk measurement tools in patients with STEMI

Authors	Sample size	ML technique	Predictors	Quality metrics		
				AUC	Sen	Sp
McNamara et al., 2016 [29]	243,440	HLR	Age, HR, SBP, circulatory arrest, CS or AHF on admission, GFR, troponin I level	0.88	—	—
Karabağ et al., 2018 [28]	1708	CR	Number of points according to SYNTAX score I, age, gender, GFR, LVEF, peripheral artery disease, COPD, bifurcation lesion of the LCA	0.92	0.92	0.8
Bessonov et al., 2021 [31]	1649	BLR	Age ≥65 years, AHF III–IV (T. Killip class III–IV), total myocardial ischemia time ≥180 min, anterior MI localization, unsuccessful PCI, SYNTAX≥16 points, Glu on admission ≥7.78 mmol/L for patients without DM 2 and Glu≥14.35 mmol/L for patients with DM 2	0.902	0.81	0.8
Hadanny et al., 2021 [32]	25,475	RF	Age, HR, MAP, AHF class (T. Killip class), Cr, Hb, Glu, total cholesterol, BMI, symptom onset to balloon time	0.78	—	—
Millo et al., 2021 [33]	346	CR	SBP, DBP, LV EDP, MAP	0.795	—	—
Tan et al., 2021 [34]	2074	MLR	Age, WBC, Hb, RBC, RDW, Glu, plasma bicarbonate and magnesium levels, peripheral arterial lesions, AFib, CS or circulatory arrest on admission, norepinephrine administration, diuresis volume	0.885	—	—
Jain et al., 2022 [35]	6165	ANN	Heart valve disease, CHF, peripheral arterial disease, coagulopathy, fluid and electrolyte disorders, CRF, hyperlipidemia, history of PCI, history of CABG, smoking, age, race, PICS, obesity, gender, and etc.	0.85	—	—
Deng et al., 2022 [36]	854	DT SVM ANN RF	AHF class (T. Killip class), ALB, CPK-MB, stent length, Cr, LVEF, WBC, LDL, symptom onset to first medical contact time, hs-CRP, troponin I level, Glu, age, Hb, Fib, and etc.	0.93	—	—
Zhao et al., 2023 [37]	8158	DT RF SVM SGB	Gender, age, RR, HR, SBP, DBP, AHF class (T. Killip class), troponin I level, impaired consciousness, hospitalization ways, reperfusion techniques, symptom onset to first medical contact time	0.85	0.85	0.76
Li et al., 2023 [38]	2414	SGB	Age, AHF class (T. Killip class), HR, SBP, BMI, increase in Cr, increase in BNP, troponin I level, CPK-MB, LA diameter, LV EDV, LCA stenosis, RCA stenosis, history of PCI, and etc.	0.913	0.845	0.858
Shakhgeldyan et al., 2024 [26]	4677	MLR RF SGB	Age, HR, SBP, AHF class (T. Killip class), Cr, LVEF, NEUT, EOS, PCT, Glu	0.9	0.843	0.838
Zhu et al., 2024 [39]	5836	EM	D-dimer, BNP, NEUT, PTT, CS, BUN, circulatory arrest, P	0.932	0.881	0.864

N o t e: AFib — atrial fibrillation, AHF — acute heart failure, ALB — albumin, ANN — artificial neural networks, AUC — area under the ROC curve, BLR — binary logistic regression, BMI — body mass index, BNP — brain natriuretic peptide, BUN — blood urea level, CABG — coronary artery bypass grafting, CHF — chronic heart failure, COPD — chronic obstructive pulmonary disease, CPK-MB — MB fraction of creatine phosphokinase, CR — Cox regression, Cr — creatinine, CRF — chronic renal failure, CS — cardiogenic shock, DBP — diastolic blood pressure, DM 2 — diabetes mellitus type 2, DT — decision tree, EM — ensemble of models, EOS — eosinophils, Fib — fibrinogen, GFR — glomerular filtration rate, Glu — blood glucose, Hb — hemoglobin, HLR — hierarchical logistic regression, HR — heart rate, hs-CRP — high-sensitivity C-reactive protein, LA — left atrium, LCA — left coronary artery, LDL — low density lipoproteins, LV EDP — left ventricular end-diastolic pressure, LV EDV — left ventricular end-diastolic volume, LVEF — left ventricular ejection fraction, MAP — mean arterial pressure, MI — myocardial infarction, ML — machine learning, MLR — multivariate logistic regression, NEUT — neutrophils, P — phosphorus in blood, PCI — percutaneous coronary intervention, PCT — thrombocrit, PICS — post-infarction cardiosclerosis, PTT — prothrombin time, RBC — erythrocytes, RCA — right coronary artery, RDW — red blood cell distribution width, RF — random forest, RR — respiratory rate, SBP — systolic blood pressure, Sen — sensitivity, SGB — stochastic gradient boosting, Sp — specificity, SVM — support vector machine, SYNTAX — Synergy between PCI with Taxus and Cardiac Surgery, WBC — leukocytes.

## “Classical” scales to predict the risk of hospital mortality in patients with STEMI

Active scientific work associated with the development of prognostic scales to stratify the risk of HM in patients with acute forms of CHD has been ongoing since the end of the last century and is caused by two main reasons: the increasing morbidity and mortality of the population from cardiovascular diseases in most countries worldwide and the intensive development of ML techniques. To develop prognostic ML models in clinical medicine, multivariate logistic regression (MLR), Cox regression (CR), random forest (RF), decision tree (DT), artificial neural networks (ANN), stochastic gradient boosting (SGB), support vector machines (SVM) and ensembles of models are most often used [62–66].

The PREDICT scale, which was one of the first scales introduced to the professional community, was developed in 1999 based on the results of the Minnesota Heart Study (MHS), which included data from 6134 patients from the acute coronary syndrome (ACS) registry, and was validated on a sample of 3570 patients with STEMI [7]. The MLR-based prognostic algorithm demonstrated acceptable accuracy in predicting HM (AUC — 0.79).

In 2000, the TIMI risk score for STEMI was developed based on the data from the InTIME II registry, which contained the results of examination and treatment of 15,078 patients with STEMI [5]. The scale included HM predictors, which were further used in other risk measurement tools and are still relevant. These predictors include the age of the patients, the T. Killip class of acute heart failure (AHF), heart rate (HR), and systolic blood pressure (SBP). The combination of these indicators with such factors as diabetes mellitus type 2 (DM 2), arterial hypertension (AH), body weight, history of angina pectoris, time to revascularization >4 h, and myocardial infarction (MI) localization allowed to prepare acceptable prognosis accuracy (AUC — 0.784); this became the basis for future research. The scale was validated with data of 3687 patients with STEMI in the TIMI-9 study, whereas the HM probability was stratified into low, intermediate, and high risk groups.

The GUSTO scale was developed in 2000 based on the results of a similarly named multicenter study that contained data of 41,021 patients with STEMI [8]. A prognostic algorithm based on CR, in addition to the age of patients, HR, MI localization, and indicators of chronic heart failure (CHF), first included an indicator of left ventricular ejection fraction (LVEF). At that, this factor in the model structure did not ensure the expected increase in the model accuracy (AUC — 0.8), which was comparable to algorithms without this predictor. The prognostic “neutrality” of the LVEF factor in this model can be explained by the fact that it lacks categorization that specifies threshold values enhancing the predictive potential [67].

The PAMI scale, introduced in 2004 and based on 4 registries of patients with STEMI, was not more accurate than the previously developed prognostic tools [9]. Predictors of the MLR prognostic model included the age of patients, AHF class (T. Killip class), heart rate, DM 2, and MI location. Indicators with a previously proven link to HM in the algorithm structure ensured its acceptable predictive accuracy, comparable to the TIMI risk score for STEMI (AUC — 0.784).

The authors of the Zwolle scale (2004) [10] were the first to draw attention to the prognostic value of such potential HM predictors as triple vessel coronary artery disease (TVCAD) and the degree of restoration of coronary blood fow according to TIMI. The combination of these factors with the indicators of age, AHF class (T. Killip class), time to myocardial revascularization >4 h, and anterior MI demonstrated excellent predictive accuracy (AUC — 0.902).

The CADILLAC scale, developed in 2005 based on analysis of data from the registry with the same name and validated on a cohort of patients from the Stent-PAMI study, combined predictors of HM and annual mortality in patients with STEMI in its structure [4]. The prognostic algorithm based on MLR included indicators of patients’ age, AHF class (T. Killip class), glomerular filtration rate (GFR), LVEF, indicators of coronary blood fow restoration according to TIMI, hematocrit (Ht), and TVCAD. GFR and Ht indicators were first applied as HM predictors and were subsequently used in other prognostic algorithms. The CADILLAC score demonstrated appropriate predictive accuracy for HM, including in patients with STEMI after percutaneous coronary intervention (PCI) (AUC — 0.83) [4].

The GRACE scale was developed in 2006 using CR and was based on the data from the similarly named international registry of patients with ACS that contains information about 43,810 patients (21,688 — a sample for training the model, 22,122 — a sample for the model validation), and was subsequently recommended for clinical use in most countries worldwide [3]. The structure of the scale included previously known HM predictors: patients’ age, HR, SBP, AHF class (T. Killip class), as well as indicators of creatinine (Cr) concentration in the blood serum, ST segment elevation, diagnostically significant increase in the level of heart-specific enzymes, and cardiac arrest at the patient’s admission to hospital.

The advantage of the GRACE scale is the combination of appropriate predictive accuracy (AUC — 0.83) and availability of predictors for HM risk stratification (low, medium, and high). The version of the scale updated in 2014 (GRACE 2.0) allows to assess the risk of mortality 1 and 3 years after ACS [11]. In a number of studies, modification of the GRACE scale was made by adding new HM risk factors to its structure [12–23]. The most accurate prognosis (AUC — 0.927) was made by a model the predictors of which included LVEF and blood leukocyte content [24].

The first Russian risk measurement tool to assess HM probability in patients with STEMI was the RECORD scale, developed in 2010 on the basis of univariate logistic regression and MLR on data from 796 ACS patients from regional medical institutions included in the similarly named register [6]. The scale structure included six predictors: patients’ age, AHF class (T. Killip class), SBP, DM 2, ST segment elevation, and hemoglobin (Hb) content in blood. HM likelihood was stratified into low-risk and high-risk groups. Available predictors and the appropriate level of predictive accuracy (AUC — 0.856) are the main advantages of the scale. Its disadvantages include the lack of validation on large independent samples. Evaluation of HM risk in patients with STEMI, which was made at the prehospital stage, was conducted using a modified RECORD algorithm with no Hb indicator in the structure, which did not reduce the predictive accuracy [25].

## New hospital mortality risk measurement tools for patients with STEMI

Improved quality of prognostic algorithms is related to the use of explainable artificial intelligence techniques, which allow to develop interpretable ML models that provide for clinical justification for the generated conclusion [26]. Their importance increases when predicting HM after emergency myocardial revascularization. This is due to the need to assess the degree of coronary damage, which is made according to SYNTAX score I (SS I), developed in 2006, and SYNTAX score II (SS II), presented in 2013 [27, 68]. The complex of SS II predictors, in addition to anatomical indicators of coronary blood fow violations, contains clinical and anamnestic data of patients: age, gender, GFR, LVEF, damage to peripheral arteries, history of chronic obstructive pulmonary disease. Recently, the number of publications in which the SS II scale is used to predict HM in patients with STEMI has increased. These include Karabağ et al. [28], where excellent predictive accuracy of HM after PCI was demonstrated (AUC — 0.92). Prognostic algorithms for HM prior to PCI were shown by McNamara et al. [29]. These algorithms, which were developed on data from the ACTION registry (243,440 patients with ACS) using hierarchical logistic regression, had equivalent accuracy in groups of patients with STEMI and NSTEMI (AUC — 0.88). Based on the prediction results, HM probability was stratified into 5 risk degrees, the lowest corresponding to 0.4%, and the highest — to 49.5%.

The interlink between HM and the effectiveness of thrombolytic therapy (TLT) was assessed using the EERIAM-HCC scale in [30]. The model was developed using an ensemble of ML techniques and demonstrated excellent predictive accuracy (AUC — 0.92). The greatest influence on HM was made by the indicator of continuous blood glucose concentration (Glu) and the categorical indicator of the QT interval >60 ms.

In [31], the authors presented a prognostic model of HM after PCI based on binary logistic regression, which was based on 7 predictors, including the duration of general myocardial ischemia and signs of PCI failure. The predictive algorithm had excellent accuracy (AUC — 0.902).

Based on analysis of data from 2782 patients with STEMI from the ACS registry (ACSIS), using the RF technique, Hadanny et al. [32] developed a prognostic model of HM after PCI, which was validated on 22,693 patients with STEMI (MINAP ACS registry). The model was based on 10 predictors, including the symptom onset to balloon time. Despite the scale structure having known factors of unfavorable outcome, it corresponded to only acceptable predictive accuracy (AUC — 0.78).

Millo et al. [33] used a cohort of 346 patients with STEMI aged over 60 years and developed the LASH score to assess the risk of HM after PCI. In this study, the authors used central hemodynamic monitoring data, including mean arterial pressure and left ventricular end-diastolic pressure after PCI in combination with SBP and DBP parameters, to model HM prognosis. When compared with the TIMI risk score for STEMI and GRACE algorithms, this scale demonstrated lower predictive accuracy (AUC — 0.881 for TIMI risk score for STEMI and 0.847 for GRACE vs 0.795 for LASH). Despite lower values of the quality metrics, this scale may be useful to assess the HM risk in patients with STEMI and cardiogenic shock, when indicators of the current hemodynamic status are of key importance for the prognosis. This approach was further supported by Tan et al. [34] based on data from 2074 patients with STEMI and NSTEMI from the eICU-CRD registry. Based on MLR, the HM prognostic model was developed for intensive care units (ICU) and validated on 1026 patients from the MIMIC-III database. The model included 14 HM predictors, 2 factors of which had the greatest impact on the end point: circulatory arrest (CA — 3.87) and the use of norepinephrine to stabilize hemodynamics (CA — 2). This model was superior in predictive accuracy to classical risk measurement scales used in ICUs (AUC — 0.885 vs 0.86 for SAPS II, 0.84 for OASIS, and 0.81 for SOFA).

Special attention should be paid to Jain et al. [35], who studied data from 6165 patients with STEMI aged 18 to 44 years. The ANN-based HM prediction model developed in this study had appropriate accuracy (AUC — 0.85) and was based on 22 predictors, first including tobacco smoking, alcohol abuse, drug addiction, and depression. Here, 2 factors were of utmost importance for the fatal event: damage to heart valves and fluid and electrolyte disorders.

Deng et al. [36] used DT, RF, SVM, and ANN techniques to build HM models on data from 854 patients with STEMI after PCI. In this study, an additional end point was the incidence of unrestored coronary blood fow after PCI. RF-based models had excellent predictive accuracy for HM (AUC — 0.93) and acceptable predictive accuracy for no-reflow (AUC — 0.78). Zhao et al. [37] used data of 8158 patients with STEMI to develop HM prognostic models based on 4 ML techniques (DT, RF, SVM, SGB). In addition to HM classical factors, hospitalization routes, reperfusion therapy techniques (primary PCI, TLT, TLT + PCI, no therapy), and symptom to first medical contact time were considered as predictors. Models based on SVM had the best accuracy for HM prediction (AUC — 0.85).

The greatest influence on the end point was made by reperfusion therapy techniques, patients’ age and SBP, and the least — by the symptom to first medical contact time and impaired consciousness signs. Reverse results were seen in [38], where the authors, based on SGB model, demonstrated that the impact of age, AHF class (T. Killip class), and SBP on HM is less significant than BMI, concentration of brain natriuretic peptide (BNP), and left atrial diameter. In this study, the predictive value of HR and Cr factors was predominant.

In [26], based on MLR, RF, and SGB, HM prognostic models were developed for various stages of medical care (before and after emergency PCI) with appropriate (AUC — 0.85) and excellent (AUC — 0.9) prediction accuracy, respectively. This study tested new techniques to identify threshold values of predictors, providing for their classification as HM risk factors and ensuring clinical justification for the prognosis results.

Zhu et al. [39] on a cohort of 5836 patients with STEMI and NSTEMI after PCI (3587 — training sample, 1196 — test sample, 1053 — validation sample) used 7 ML techniques to develop HM models with excellent prediction accuracy. The model based on an ensemble meta-algorithm (Bagging) had the highest accuracy (AUC — 0.932) and included 8 predictors, where D-dimer, BNP, and blood phosphorus concentration have not been previously used in the analyzed algorithms. It is noteworthy that D-dimer and BNP had the greatest impact on HM, and the impact of neutrophils and prothrombin index to the end point was comparable to the cardiogenic shock factor.

## Discussion

In recent years, predictive analysis techniques have been widely used in clinical medicine, which is confirmed by the constantly growing number of scientific researches on the issue [69–80]. This approach is of particular relevance in life-threatening conditions, including STEMI. The systematic review provides an analysis of publications showing the evolution of HM prediction techniques in STEMI over the past 25 years. The majority of the “classical” scales were developed and validated on large samples of patients in the first decade of this century (see Table 1). At that, 3 of them (PREDICT, GRACE, RECORD) were developed based on data from the similarly called registries of patients with ACS, and 5 scales (GUSTO, TIMI risk score for STEMI, Zwolle, PAMI, and CADILLAC) — based on data from registries of patients with STEMI. The structure of the majority of the analyzed scales included HM predictors, which were considered referential (age of patients, AHF class (T. Killip class), HR, and SBP). The CADILLAC and Zwolle scales also include TVCAD and TIMI indicators, which are associated with a more accurate stratification of HM risk after PCI. Laboratory indicators Cr, GFR, Ht, and Hb add up to the list of predictors in the PREDICT, CADILLAC, GRACE, and RECORD scales, and LVEF indicators and time to myocardial revascularization >4 h — in the TIMI risk score for STEMI and Zwolle scales. According to the AUC values classifier [81], only the Zwolle scale had excellent predictive accuracy (AUC≥0.9), the GRACE, CADILLAC, GUSTO, and RECORD scales have appropriate predictive accuracy (0.8≤AUC<0.9), whereas PREDICT, TIMI risk score for STEMI and PAMI have acceptable predictive accuracy (0.7≤AUC<0.8). It is known that the best quality of predictive models is most often demonstrated in the populations from which the original data were received [82]. At that, many studies demonstrated that the GRACE and CADILLAC scales used on external samples had higher predictive accuracy than other classical scales [83, 84]. It should be noted that all analyzed scales were developed using the following basic ML techniques: MLR and CR. Their advantage is the transparency of predictive decisions, and the disadvantage relates to considering only linear relations between predictors and the end point of the study, which limits their predictive potential.

Currently, an active search for new predictors of STEMI-associated adverse events is ongoing. In various publications, newly identified HM predictors for this category of patients are related to signs of comorbidity, clinical, biochemical, and hematological indicators of inflammatory response, metabolic status parameters, heart chamber volumes, atrial fibrillation, BNP, D-dimer, and etc. Due to the prevailing strategy of myocardial revascularization by means of PCI, the need to develop HM risk measurement tools after PCI is growing. According to literature, fatal outcomes after emergency PCI are recorded in 4–7% of patients with STEMI, which makes the issue of HM risk stratification relevant [62]. HM predictors after PCI most often include signs of failure according to TIMI — 0–2 (slow-reflow and no-reflow phenomena), criteria of coronary lesions according to SS I or SS II, hemodynamic parameters, blood glucose levels, and etc. [85–98].

To increase the information value of predictors and develop interpretable ML models, explainable artificial intelligence techniques have been used in recent years. These techniques include identification of predictors’ threshold values, deviation from which increases their predictive value and allows their classification as risk factors for adverse events [67]. A new approach in ML model development is phenotyping of risk factors and ranking of specific predictors per the intensity of their influence on the end point of the study [26, 99–102]. The analysis of recent publications demonstrated that the majority of the current predictive models were developed using modern ML techniques (RF, SVM, DT, SGB, ANN, ensembles of models), which in most cases have appropriate or excellent accuracy (see Table 2). Their advantage is the ability to identify hidden or non-obvious patterns, as well as to get new knowledge from big data. An important area of risk measurement in STEMI is still the development of HM prognostic models for patients in the ICU due to the disease complications. The review mentions publications with examples of such algorithms, the predictors of which include invasive indicators of cardiohemodynamics, indicators of oxygen delivery and consumption, blood lactate concentration, administration of vasoactive drugs, and etc. These models were superior in accuracy to classical emergency risk scales (APACHE II, SAPS, SI) [40-43].

## Conclusion

The analysis of scientific literature points to a growing interest of researchers in improvement of prognostic technologies that provide reliable risk stratification of hospital mortality in STEMI. This issue is associated with development of interpretable ML models that can explain generated conclusions, which contributed to building trust of doctors. Here, an important task is implementation of prognostic models of adverse events in medical decision support systems, which provide additional information required to assess the risks of hospital mortality in daily clinical practice. A necessary condition for evolution of prognostic technologies for hospital mortality is also development and constant update of regional and national registers of patients with STEMI, which take into account specifics of resource support for cardiological service.
